# Creation and resolution of non-B-DNA structural impediments during replication

**DOI:** 10.1080/10409238.2022.2121803

**Published:** 2022-09-28

**Authors:** Christopher Mellor, Consuelo Perez, Julian E. Sale

**Affiliations:** Division of Protein & Nucleic Acid Chemistry, MRC Laboratory of Molecular Biology, Cambridge, UK

**Keywords:** DNA replication, DNA secondary structure, G quadruplex, DNA helicases, DNA polymerases

## Abstract

During replication, folding of the DNA template into non-B-form secondary structures provides one of the most abundant impediments to the smooth progression of the replisome. The core replisome collaborates with multiple accessory factors to ensure timely and accurate duplication of the genome and epigenome. Here, we discuss the forces that drive non-B structure formation and the evidence that secondary structures are a significant and frequent source of replication stress that must be actively countered. Taking advantage of recent advances in the molecular and structural biology of the yeast and human replisomes, we examine how structures form and how they may be sensed and resolved during replication.

## Introduction

**Figure 1:**
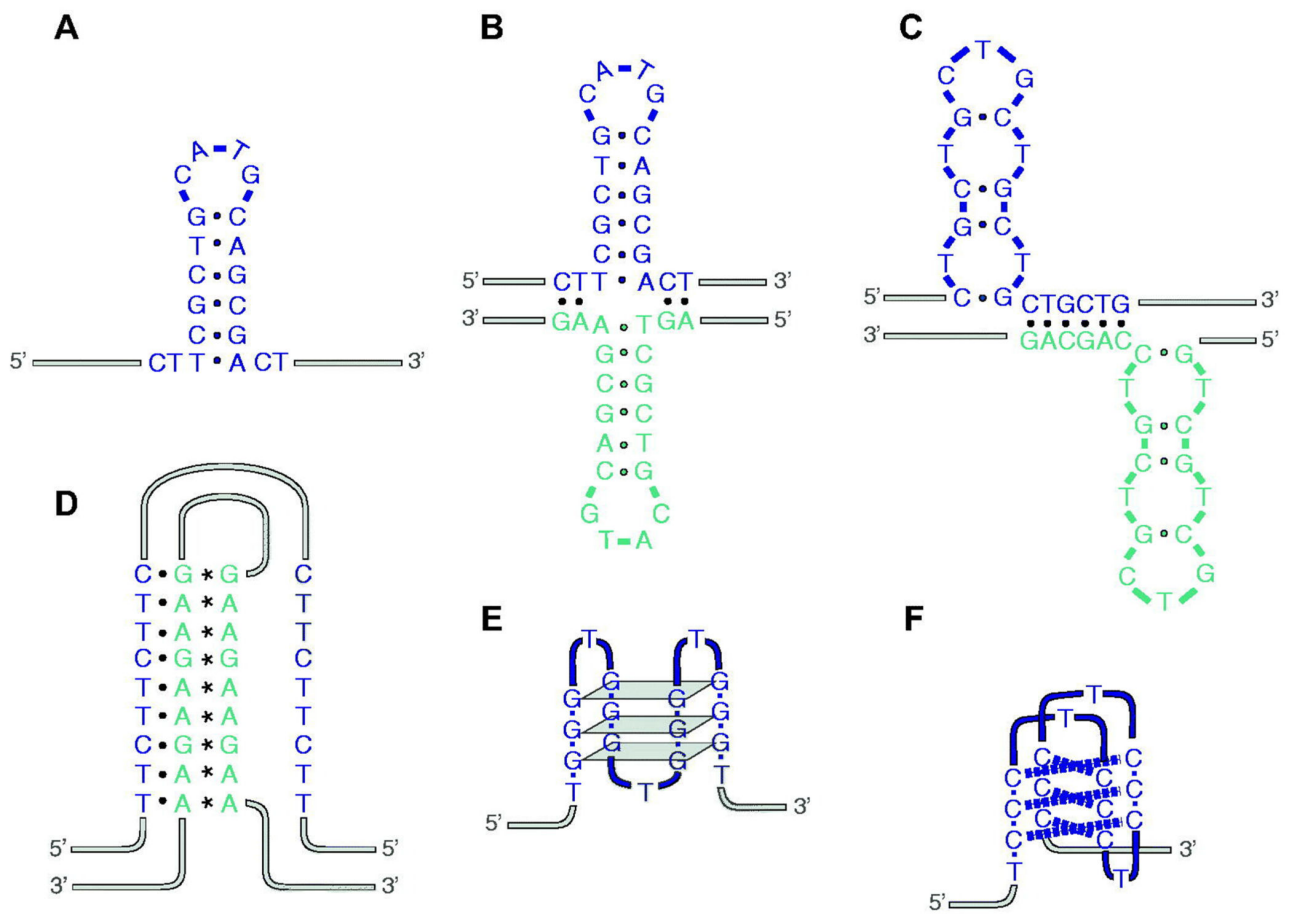
Key non-B-form DNA structures. (A) Hairpin. (B) Cruciform. (C) Slipped strand DNA (S-DNA). (D) Triplex or H-DNA. (E) G quadruplex (G4). (F) I-motif. The protonation of one of the dCs in each pair, required for the formation of these structures, is not shown. A color version of this figure is available online.

The predominant conformation of the DNA within cells is the canonical right-handed double helix, or B-DNA. Under certain circumstances, DNA can adopt a range of non-B-form secondary structures (Figure 1). The formation of these alternate secondary structures is primarily dependent on local sequence features including symmetry, repetitive tracts and GC content. However, whether a specific sequence with non-B-form structural potential adopts a non-canonical secondary structure in a cell is determined by many additional factors including DNA unwinding, the superhelical status of the DNA, the presence of additional nucleic acid strands such as RNA, and protein interactions. The formation of non-B secondary structure in DNA has profound consequences for the smooth execution of the normal transactions that take place in the genome, particularly replication, transcription and repair, since they can block the translocation of critical enzymes, notably the helicases and polymerases, that carry out these reactions. In addition, replication and transcription create negative supercoiling and single-stranded DNA (ssDNA), which both favor non-canonical structure formation.

Considering that nearly half of the human genome consists of low complexity, often repetitive sequences (Jurka et al. 2007; Treangen and Salzberg 2011), the opportunity for structure formation and replication stalling is significant. Despite the potentially positive roles of secondary structure in genome function (reviewed in Maizels and Gray 2013; Rhodes and Lipps 2015; Murat and Balasubramanian 2014; Varshney et al. 2020), it is becoming clear quite how many of these sequences have the capacity to perturb replication, making DNA itself one of the greatest impediments to its own replication. We will focus on recent developments in genetic, biochemical and structural analysis of DNA replication that provide insights into several key questions: What drives a sequence with non-B form structural potential to become a replication impediment? How does the replisome encounter and generate DNA structural impediments? How does the replisome respond to ensure smooth progression of replication, avoiding genetic and epigenetic instability?

## The non-B-form genome

The most important types of non-B conformations of DNA are summarized in Figure 1. In general, non-B-form DNA is more likely to arise in the repetitive or low complexity sequences that make up over 50% of the human genome (Treangen and Salzberg 2011). However, the conformation adopted is strongly influenced by the precise sequence composition which determines the nature of the base pairing that stabilizes the structure. Thus, hairpins in ssDNA DNA, or cruciform structures in double-stranded DNA, form in inverted repeats and are stabilized by complementary Watson-Crick base pairing (Lilley 1980; Panayotatos and Wells 1981). The triple-helical conformation known as hinged-DNA (H-DNA) may form in mirror repeats, but it does so only when there are homopurine-homopyrimidine tracts that allow the Hoogsteen base-pairing needed to stabilize the structure (Mirkin et al. 1987). Direct tandem repeats have the potential to form a wide variety of conformations, again depending on the base composition: alternating purine and pyrimidine runs can flip to a left-handed double helical conformation, termed Z-DNA (Wang et al. 1979). Slipped-strand DNA or S-DNA is formed when complementary repeats mispair following denaturation/renaturation cycles, resulting in combinations of stretches of double stranded DNA and single-stranded loops (Coggins and O’Prey 1989; Kovtun and McMurray 2008). A-rich repeats containing tracts of 4 – 6 adenines can lead to the formation of intrinsically bent DNA (Koo et al. 1986). However, while the conformational constraints imposed by A-tract DNA may play important regulatory roles, for instance in transcription, it is unclear that it has any direct impact on DNA replication because of these physical properties. Rather, poly dA tracts are vulnerable to replication fork collapse because of their relatively poor ability to interact with the single strand binding protein RPA, which protects ssDNA during replication (Tubbs et al. 2018). Sequences containing tandemly arranged tracks of guanines can fold into a structure known as a G-quadruplex (G4). G4 secondary structures are driven by Hoogsteen hydrogen bonding of guanine quartets, stacked on top each other through p-p interactions (Gellert et al. 1962). G4s are conformationally diverse (Lightfoot et al. 2019), which is driven by the number of tetrads and the composition and length of the loop sequences. They can be formed from four separate strands (tetramolecular), two strands (bimolecular) or from a single strand of DNA or RNA (unimolecular). The latter two forms are likely the most important in terms of impeding DNA replication as they can arise in the template alone or template in complex with another nucleic acid strand, such as a nascent transcript. Conversely, C-rich sequences have the potential to form i-motifs, which comprise intercalated dC: protonated dC pairs. The requirement for protonation was originally considered to exclude the possibility of physiologically relevant formation of these structures at cellular pH. Thus, the i-motif forming human telomeric C-rich strand (C3TA2)4 can stall DNA synthesis by the Klenow fragment at pH 6.0, but not at pH 7.0 (Takahashi et al. 2017). However, it is now clear that i-motifs can form in cells (Zeraati et al. 2018) and certain i-motif sequences such as ATCCTC and ACCCGG do indeed form at physiological pH *in vitro* and can stall DNA synthesis (Murat et al. 2020).

In many cases, the sequence dependence of DNA secondary structures allows computational prediction of where in the genome each has the potential to form (e.g. Hon et al. 2013; Bedrat et al. 2016). While these predictions have been applied to many non-B DNA conformations, in some cases the prediction is complicated by the fact that physiologically relevant structures can require the presence of an additional strand of nucleic acid, e.g. RNA, to form (Duquette et al. 2004; Šviković et al. 2019; Miglietta et al. 2020). Sequence-based prediction is probably most advanced for G quadruplexes (G4s) (Puig Lombardi and Londoño-Vallejo 2020). Early estimates of the G4-forming potential of the human genome based on the consensus sequence G_3-5_N_1-7_G_3-5_N_1-7_G_3-5_N_1-7_G_3-5_ suggested some ~350,000 sites (Huppert and Balasubramanian 2005; Todd et al. 2005). However, these early approaches underestimated the polymorphic nature of G4-forming sequences and clearly missed sequences that could be shown biophysically to form G4s *in vitro*. More recent methods using sliding windows, scoring systems or machine learning are better able to pick up unusual G4-forming sequences (Bedrat et al. 2016; reviewed in Puig Lombardi and Londoño-Vallejo 2020). Currently, the most definitive maps of G4-forming potential have been generated by a genome-wide high throughput sequencing-based approach, which leveraged the ability of K^+^ ions or ligands to control G4 formation in the template of Illumina sequencing reactions. This method identified ~700,000 DNA G4 sites in the human genome (Chambers et al. 2015), doubling the number of sites estimated by the early algorithms, and has been extended to a further dozen species (Marsico et al. 2019).

However, showing where secondary structure *can* form does not indicate where it *does* form in a cellular context. It is clear from efforts to detect G4s in human cells using a G4-binding antibody (BG4), that only a subset of sequences is likely to be folded at a given time and that folding is more likely during S phase (Biffi et al. 2013). Further, genome-wide mapping of folded G4s using BG4 picks up only a small fraction of potential quadruplex-forming sequences. Those that were detected were more likely to be found in certain genomic environments, such as open chromatin (Hänsel-Hertsch et al. 2016). Nonetheless, functional assays to detect the impact of non-B-form structures on DNA replication, discussed further below, demonstrate that specific structures are likely to form very frequently during DNA replication (Schiavone et al. 2016; Guilbaud et al. 2017; Lee et al. 2021). This discrepancy may be explained by structures being only relatively short lived, a notion that is supported by recent *in vivo* single molecule probing of G4 formation (Di Antonio et al. 2020). However, the half-life of different forms of structure in different cellular contexts remains relatively poorly understood and may well vary considerably.

## Factors driving secondary structure formation *in vivo*

Transition of susceptible DNA sequences from B-form to alternative conformations capable of impeding replication requires the Watson-Crick DNA duplex to be melted. Thus, understanding the drivers of secondary structure formation requires understanding of the mechanisms that generate the conditions for such structures to form, particularly how sufficiently long tracts of melted duplex arise.

### DNA breathing

The DNA double helix is not static. Even at physiological temperatures fluctuations in the conformation of the DNA duplex can result in local melting of base pairs to create single stranded regions known as DNA bubbles. This phenomenon is known as DNA breathing (Guéron et al. 1987; Metzler and Ambjörnsson 2005). Below the melting temperature of the DNA, bubbles are uncommon, but once formed can spread as the free energy required for disrupting Watson-Crick base pairing is rather lower than that required for overcoming the initial barrier to bubble formation (Ambjörnsson et al. 2007). While DNA breathing does appear to be important for the initial interaction of some DNA binding proteins (e.g. Alexandrov et al. 2012), it is less clear that thermal denaturation on its own it is sufficient to support significant secondary structure formation in cells. However, as discussed below, negative supercoiling is likely to provide significant additional drive to denaturation even under conditions in which normal base pairing is energetically favored (Hsieh and Wang 1975; Benham 1979).

### Supercoiling induced denaturation

**Figure 2:**
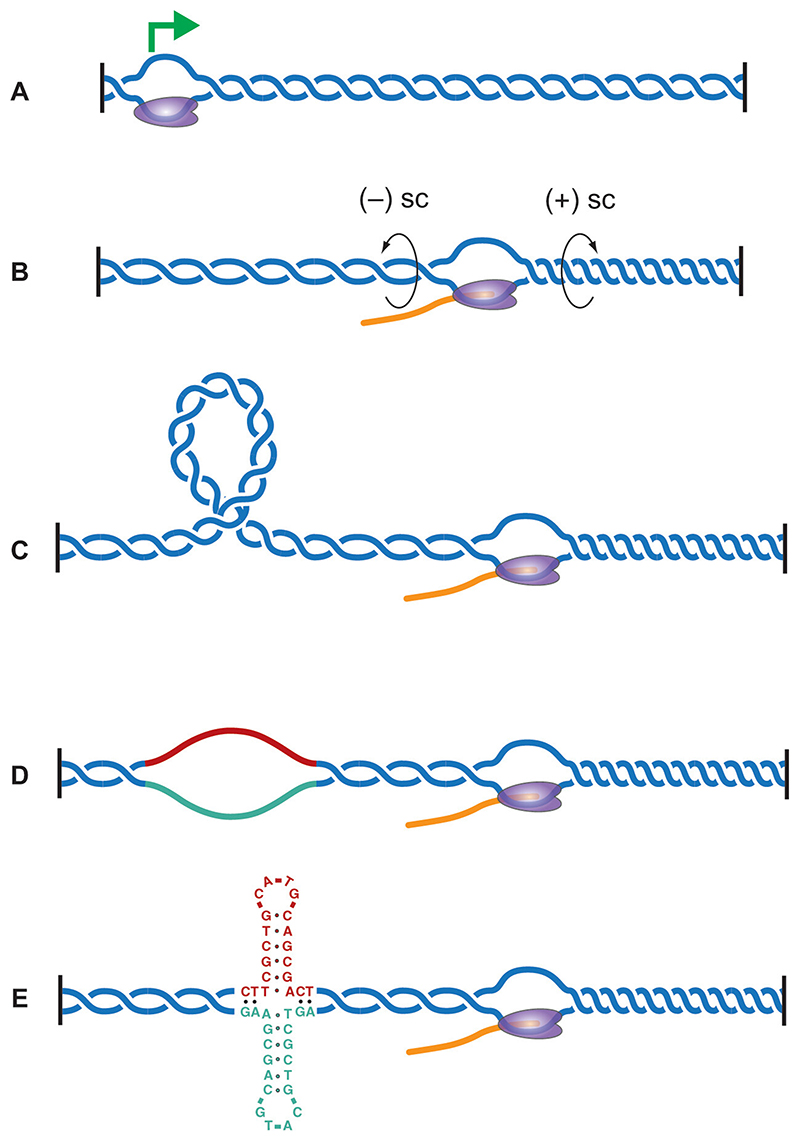
Formation of DNA secondary structures driven by transcription-induced supercoiling. (A) A transcribed segment of DNA bounded by constraints (black bars) that prevent propagation of supercoiling. RNA polymerase (purple) is shown loaded at a transcription start site. (B) Positive twist (+sc) is generated ahead and negative twist (-sc) is generated in the wake of the advancing polymerase. This is countered by the action of topoisomerases. However, in very transcriptionally active genes, these enzymes may not be able to keep pace leading to a net build-up of supercoiling. This may be converted into writhe (C) or can lead to denaturation of the double helix creating a single stranded bubble (D). If such bubbles occur in a sequence that has secondary structure forming potential (red & green segment), this can promote non-B-DNA structure formation, illustrated here by a cruciform forming in an inverted repeat sequence (E). A color version of this figure is available online.

DNA supercoiling refers to the over- or under-winding of the DNA helix. It arises as a consequence of the activity of helicases, DNA and RNA polymerases and, to a lesser extent, of chromatin remodeler and DNA repair complexes. The transcription machinery is a major source of supercoiling in eukaryotic genomes with the tracking of the RNA polymerase along the DNA helix building up positive supercoiling ahead of the polymerase and negative supercoiling behind (Liu and Wang 1987; Ma and Wang 2014 and Figure 2).

Underwound or negatively supercoiled DNA weakens Watson-Crick base paring and facilitates DNA strand separation creating denatured ‘bubbles’ within the duplex (Figure 2). These bubbles absorb supercoiling energy by allowing a reduction in the number negative superhelical turns in the DNA segment. Although, denaturation domains in negatively supercoiled DNA were predicted, and demonstrated, to favor alternative structure formation many years ago (Vinograd et al. 1965; Hsieh and Wang 1975; Giaever et al. 1988), there has been continuing debate over the extent to which supercoiling-induced denaturation alone is sufficient to drive structure formation.

In plasmids, negative supercoiling can promote formation of Z-DNA (Azorin et al. 1983; Nordheim and Rich 1983), H-DNA (Mirkin et al. 1987; Kohwi and Kohwi-Shigematsu 1988; Vojtísková et al. 1988; Potaman et al. 2004) and cruciforms (Panayotatos and Wells 1981; Murchie and Lilley 1992) in susceptible sequences and can be explained in terms of the effect of the local DNA unwinding caused by the formation of the structure on the overall topology of the plasmid (Murchie and Lilley 1992).

Plasmids harboring sequences susceptible to non-B structure formation have also been employed as reporters for supercoiling in cells. This approach has allowed the demonstration that transcription can generate sufficient free energy in the negatively supercoiled DNA behind RNAPII to drive the formation of cruciform structures in inverted repeats (Dayn et al. 1992; Jude et al. 2013) and ssDNA bubbles and non-B structure formation in a transcriptional regulatory element from the human *c-MYC* locus termed the supercoil-sensitive far upstream element (FUSE) (Kouzine et al. 2004; 2008).

*In vivo*, mapping of supercoiling using detection of intercalated psoralen, which preferentially interacts with negatively supercoiled DNA, shows that significant negative supercoiling is found at rather localized sites in the genome (Kouzine et al. 2013; Naughton et al. 2013) and diffuses along the DNA fiber for relatively short distances, up to a few kb (Krasilnikov et al. 1999; Kouzine et al. 2004; 2013; Zhang et al. 2013). It is likely that these sites reflect a combination of failure of topoisomerases to keep pace with bursts of RNAPII activity in the context of local constraints on chromatin that prevent supercoiling from being dissipated (Kouzine et al. 2013; Schalbetter et al. 2015; Minchell et al. 2020). Exploiting the observation that permanganate mapping of ssDNA yields specific profiles when the ssDNA is formed in the context of different non-B form DNA structures, Kouzine et al. were able to demonstrate a genome wide correlation between supercoiling and ssDNA/non-B form structure formation, providing further evidence that intracellular supercoiling may be the main driver of many unusual DNA structures *in vivo* (Kouzine et al. 2017).

Is the energy held in negatively supercoiled DNA able to drive formation of all non-B structures equally? Probably not. Further, the link between supercoiling and non-B structure formation is additionally complicated by interactions between sequences and the conformations they can adopt. The ability of Z-DNA to absorb the energy of negative supercoiling can, in turn, limit the formation slipped strand DNA, exemplified by the (CCTG):(CAGG) repeats found in the *ZNF9* locus in myotonic dystrophy type 2 (Edwards et al. 2009). However, the evidence that supercoiling is sufficient to readily induce G4 formation is less clear cut. While studies have suggested that propagation of transcription-induced supercoiling can induce G4 formation (Zhang et al. 2013), in other experimental systems G4 formation has required the presence of a stabilizing structure of the C-rich strand, such as an R-loop (Duquette et al. 2004), i-motif (Sun and Hurley 2009) or other complex structure (Voloshin et al. 1992), or a peptide nucleic acid (Onyshchenko et al. 2009). In the absence of such assistance, detailed analyses suggest that G4s form poorly or not at all in supercoiled plasmid DNA (Sekibo and Fox 2017) or in the genome (Kouzine et al. 2017).

### Indirect contributions of transcription to promoting non-B structure formation: RNA:DNA hybrids and R-loops

As noted above, the folding of many secondary structures that can form in ssDNA are promoted when the complementary DNA strand is sequestered. An important mechanism by which transcription can contribute to this is through the formation of RNA:DNA hybrids, in which the nascent mRNA hybridizes to its DNA template. When the non-template DNA strand is displaced, the structure is termed an R-loop. The formation and consequences of RNA:DNA hybrids and R-loops has been extensively reviewed elsewhere (e.g. García-Muse and Aguilera 2019). An R-loop provides an ideal opportunity for secondary structure formation within the displaced strand (Duquette et al. 2004). Indeed, it is likely that RNA:DNA hybrids and the DNA secondary structures reinforce each other to varying extents in a manner that depends on the nature of the structure. In the *BU-1* locus of DT40 cells (see section below ‘*Epigenetic instability arising from non-B DNA structure-dependent fork stalling*’), the ability of a short triplex-forming sequence to cause a replication impediment is entirely dependent on RNA:DNA hybrid formation, while a G4 impediment exhibits much less dependence on an RNA:DNA hybrid (Šviković et al. 2019). Thus, factors that remove R-loops, such as RNaseH1 and helicases such as senataxin (Skourti-Stathaki et al. 2011) can exert an indirect destabilizing effect on a DNA secondary structure through RNA:DNA hybrid removal, as well as, in the case of some helicases like Pif1 (Chib et al. 2016), playing a role in unwinding the secondary structures themselves. It is worth noting, however, that the interaction between R-loops and non-B DNA structure folding is more complex than a simple strand sequestration model as R-loop formation, of course itself also a non-B DNA structure, can relieve superhelical stress in underwound DNA (Stolz et al. 2019; Chedin and Benham 2020) and could therefore also potentially counter or alter the nature of the structures formed.

Several other cellular processes can generate ssDNA DNA and hence potentially promote formation of non-B structures for example excision repair and DNA end resection, which will not be discussed further here. However, of particular relevance to this review is the action of the replisome itself, which we consider in the next section.

## Secondary structures as replication impediments

### In vitro stalling of DNA synthesis by secondary structures

Early studies of DNA replication using the SV40 system demonstrated that certain DNA sequences could specify the termination of replication (Tapper and DePamphilis 1980; Germino and Bastia 1981). Subsequent work with purified DNA polymerases revealed that the sequence of the DNA template itself was sufficient to create polymerase pause sites, which was proposed to be due to local secondary structure formation (Kaguni and Clayton 1982; Weaver and DePamphilis 1982; Hillebrand and Beattie 1985; Abbotts et al. 1988; Fry and Loeb 1992). This idea was strongly supported by the demonstration of a potassium ion-dependent DNA synthesis pause site in the chicken β-globin promoter (Woodford et al. 1994), which was subsequently shown to fold into a G4 (Howell et al. 1996). More recently, DNA polymerase inhibition by secondary structure has been harnessed in high-throughput sequencing experiments to identify structured DNA-forming sequences (Chambers et al. 2015; Sawaya et al. 2015; Murat et al. 2020). However, polymerase stalling by secondary structure is not all or nothing. We have recently found that the kinetics of polymerization by a simple replicative DNA polymerase as it traverses structured DNA contains sufficient information to classify the structures adopted by short tandem repeat sequences (Murat et al. 2020).

### Genetic instability induced by replication of non-B DNA structures

There is long standing evidence that non-B DNA structure-forming sequences are associated with genetic instability (reviewed in Javadekar and Raghavan 2015; Kaushal and Freudenreich 2019), much deriving from the study of the human repeat expansion disorders (reviewed in Khristich and Mirkin 2020).

Translocation and/or deletion breakpoints in cancers are associated with a wide range of DNA structures including G4s (De and Michor 2011; Katapadi et al. 2012; Bacolla et al. 2016; 2019), H-DNA (Bacolla et al. 2016; Zhao et al. 2018), Z-DNA (Bacolla et al. 2016) and cruciform-forming inverted repeats (Lu et al. 2015; Bacolla et al. 2016). Damage and mutagenesis at such sites can be replication dependent or independent. The latter group of replication-independent mechanisms have been the subject of recent reviews (e.g. Khristich and Mirkin 2020; McKinney et al. 2020b) and will not be covered further here. However, it is worth noting that structure-specific cleavage and the consequent engagement of homologous recombination and end-joining double strand break repair pathways (Lu et al. 2015; Zhao et al. 2018; McKinney et al. 2020a; Polleys and Freudenreich 2021) can pose a particular problem if deployed at the replication fork and can result in significant genetic instability in the context of highly repetitive sequences (reviewed in Brown and Freudenreich 2021).

Introduction of structure-forming tandem repeat sequences into a range of model systems, particularly plasmids and budding yeast, has provided firm evidence that genetic instability at such sequences can indeed arise as a direct consequence of their replication. Length stability of structure-forming repeats is modulated by the presence of a replication origin, the distance between the repeat and the origin, and the orientation of the repeat relative to the origin (Maurer et al. 1996; Cleary et al. 2002; Dere et al. 2004; Nichol Edamura et al. 2005; Pelletier et al. 2005; Rindler et al. 2006; Liu et al. 2007; 2010).

Replication-dependent instability is also seen at cruciform-forming short inverted repeats, (Lu et al. 2015) and in G4-forming long microsatellite arrays (Lopes et al. 2011; Piazza et al. 2012). In the latter case, the link to structure formation is emphasized by the amplification of instability following G4 stabilization with a ligand or following deletion of factors that suppress G4 formation (Lopes et al. 2011; Piazza et al. 2012; Maestroni et al. 2020). Likewise, G-tracts and G4-forming sequences induce genetic instability in C. elegans lacking the FANCJ helicase homologue DOG-1 and this is dependent on replication (Cheung et al. 2002; Youds et al. 2006; Kruisselbrink et al. 2008; Koole et al. 2014).

There is evidence that the composition of the replisome can also influence the chance of genetic instability at a structure-forming sequence. During the very early DNA synthesis after replication origin firing (Aria and Yeeles 2018; Garbacz et al. 2018) and during recombination-mediated fork restart (Miyabe et al. 2015; Naiman et al. 2021), Pol δ is deployed on both leading and lagging strands. Although the consequences for genome stability of δ/δ replication forks during origin firing are not yet clear, in the context of fork restart several studies have shown these forks to be more error prone (Iraqui et al. 2012; Mizuno et al. 2013; Jalan et al. 2019). Recent work has extended this concept to show that long-travelling δ/δ forks formed after encounter with a programmed fork barrier up to 7 kb distant have a greater propensity to expand CAG/CTG repeats (Gold et al. 2021).

Unstable repeat sequences can also induce break-induced replication (Kononenko et al. 2018) in which leading strand replication is driven by a migrating D-loop maintained by Pol δ and PIF1 (Wilson et al. 2013). This is distinct from the coupled, semi-consevative replication of the δ/δ forks discussed above but is also significantly more mutagenic than normal replication (Costantino et al. 2014; Vasan et al. 2014). The ability of repeat sequences to increase mutagenesis kilobases away (Shah et al. 2012), is known as repeat-induced mutagenesis (RIM). RIM induced by the triplex-forming (GAA)_n_ repeats associated with Friedreich’s ataxia (Frank-Kamenetskii and Mirkin 1995; Ohshima et al. 1998; Chandok et al. 2012) is particularly well studied. In Friedreich’s Ataxia patients, the region flanking the expanded GAA repeat in the *FXN* locus exhibits increased rates of point mutation (Bidichandani et al. 1999). The phenomenon can also be modeled in yeast (Shishkin et al. 2009) with GAA repeats introduced into the yeast genome increasing mutagenesis up to 10 kb away from the locus, including point mutations (Shah et al. 2012; Saini et al. 2013; Tang et al. 2013), deletions (Saini et al. 2013) and gross chromosomal rearrangements (McGinty et al. 2017). Defects in the replicative polymerases, Pol ε or Pol δ, reveal or exacerbate RIM (Shah et al. 2012; Tang et al. 2013; Kononenko et al. 2018), which appears to be structure independent, being observed up to 3 kb from a (CGG)n repeat capable of forming G4s in human cells (Kononenko et al. 2018), and either side of interstitial telomere sequences (Aksenova et al. 2013) and around various inverted repeats capable of forming hairpins and cruciforms in yeast (Saini et al. 2013).

### Epigenetic instability arising from non-B-DNA structure-dependent fork stalling

The flow of histone proteins from the parental to daughter DNA strands is normally very tightly coupled, with displaced histones being redeposited on the daughter strands as soon as sufficient double stranded DNA has emerged behind the polymerases. The coupling of histone displacement and deposition is not only important to ensure the daughter strands are fully chromatinised but is also vital for the propagation of post-translational modifications of the histone tails, which in turn contribute to the maintenance of transcriptional states. By maintaining the register of the modified histones with the underlying DNA sequence, the parental histones can act as a template for marking new histones introduced to maintain nucleosome density (reviewed in Stewart-Morgan et al. 2020).

**Figure 3:**
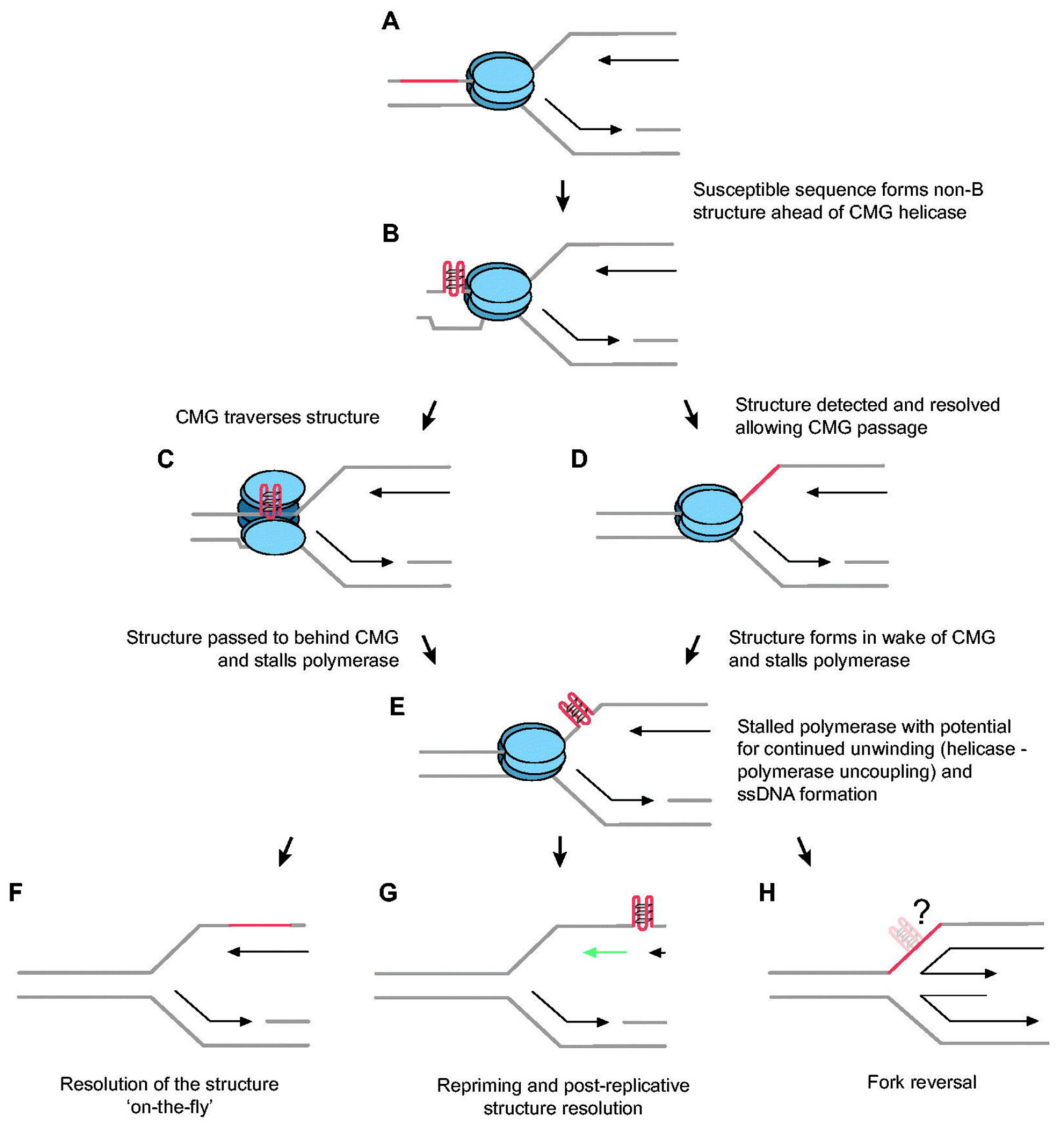
Replication fork responses to non-B DNA structures. (A) Unperturbed replication. The CMG helicase (blue) unwinds a sequence with non-B-DNA structural potential (orange). For clarity, other components of the replisome are not shown and only a leading strand impediment is considered. (B) If the sequence forms a non-B-DNA structure (here a G4) ahead of the CMG helicase, it may stall it. This can be resolved either by (C) helicase remodeling and traverse of the structure or (D) detection and resolution of the structure ahead of the fork allowing the CMG helicase to pass. In both cases, the structure now presents and obstacle to the replicative polymerase, which can lead to helicase-polymerase uncoupling due to continued activity of the helicase with consequent exposure of more ssDNA DNA (E). This can be resolved by structure resolution ‘on-the-fly’ allowing continued DNA synthesis (F) or it can lead to repriming, allowing continued coupled DNA synthesis while the structure is resolved in a post-replicative gap (G). Structures also appear to be able to induce fork reversal (H), although the significance of this for their resolution remains unclear. It is possible that the action of fork reversal helicases could lead indirectly to structure dissolution. A color version of this figure is available online.

Impediments that stall DNA synthesis can lead to helicase-polymerase uncoupling within the replisome (see Figure 3). This exposes ssDNA, which is potentially vulnerable to damage, and interrupts the normal flow of histone proteins from the parental to daughter DNA strands. The DNA synthesis at post-replicative gaps left following an episode of fork uncoupling does not necessarily have access to the usual supply of correctly modified parental nucleosomes and thus the information carried in the modifications is lost. This loss of epigenetic information can lead to changes in the level and stability of gene expression (Šviković and Sale 2017). For scattered DNA damage, detecting such changes and causally linking them to interrupted fork progression is extremely challenging. However, interruptions of DNA synthesis at secondary structures occurs recurrently at predictable locations allowing epigenetic changes to be readily observed.

This phenomenon has been studied most intensively in the avian cell line DT40 (Sarkies et al.2010;Schiavone et al. 2014). Genes containing natural or engineered non-B-DNA secondary structure forming sequences near to their transcription start site exhibit localized loss of parental histone marks and stochastic, replication-dependent changes in gene expression under conditions in which replication of the structure-forming sequence is impaired. *BU-1* encodes a cell surface lectin in avian B cells and contains a strong natural G4-forming sequence in its second intron, 3.5 kb downstream of the transcription start site. Its expression becomes unstable when enzymes involved in DNA secondary structure dissolution are deleted, for instance the Y-family polymerase REV1 or specialized helicases like FANCJ or DDX11 (Sarkies et al. 2010; 2012; Schiavone et al. 2014; Lerner et al. 2020), or when the structure is stabilized by a ligand (Guilbaud et al. 2017). Further supporting the link with replication, agents that globally impair DNA synthesis, such as hydroxyurea and aphidicolin, potentiate G4-dependent instability of *BU-1* expression (Papadopoulou et al. 2015). The slow replication with extensive helicase-polymerase uncoupling (Alvino et al. 2007), likely increases the opportunity for structure formation, a hypothesis recently supported by direct observation with super-resolution microscopy of enhanced G4 formation within replisomes of aphidicolin-treated cells (Lee et al. 2021). Collectively, these data provide strong evidence that DNA secondary structure interrupts replication and that, on the leading strand, this can lead to the formation of transient uncoupling events extending for several kilobases. Indeed, loci like *BU-1* provide a sensitive assay for loss of processive replication at a secondary structure-forming sequence by ‘recording’stalling events inthe form of epigenetic changes in a dividing cell population (Guilbaud and Sale 2021). Crucially, the prominent role of PrimPol-dependent repriming in suppressing *BU-1* expression instability, discussed further below, reveals how extraordinarily frequent secondary structure formation is during apparently unperturbed replication (Schiavone et al. 2016; Šviković et al. 2019).

Evidence of the more general nature of this complex interplay between DNA replication, non-B DNA structures and chromatin inheritance is emerging from studies of flowering regulation by floral suppressor gene *FLC* in *Arabidopsis*. One of the regulatory pathways which acts on the *FLC* locus is the autonomous floral pathway, which promotes rapid flowering in *Arabidopsis* accessions such as Col-0. Mutations within this pathway lead to a late flowering phenotype (Wu et al. 2020). At the heart of the pathway are the RNA binding protein FCA and the cleavage and polyadenylation specificity factor (CPSF) complex component FY. These proteins have widespread roles in regulating alternate polyadenylation in *Arabidopsis* (Yu et al. 2019) but are particularly well studied in the context of the autonomous floral pathway, through their roles in an antisense-mediated chromatin silencing pathway at the *FLC* gene (Wu et al. 2016). At the *FLC* locus, the 3′ processing activity of FY and FCA acts to resolve an R-loop involving the antisense *COOLAIR* transcript. This is physically linked to chromatin silencing through an interaction with the H3K4me1 demethylase FLD via the SET domain protein SDG26 (Fang et al. 2020). Loss of this 3′ end processing activity leads to perturbed fork progression in FY or FCA-deficient *Arabidopsis* (Baxter et al. 2021), similar to that observed in mammalian cells (Teloni et al. 2019). Transplantation of the 3′ end of the FLC locus, which contains G4 and H-DNA forming sequences, into the *BU-1* locus leads to R-loop dependent *BU-1* expression instability when the template strand is replicated as the leading strand in PrimPol-deficient DT40 (Baxter et al. 2021). These observations gave rise to a model in which an R-loop formed by anti-sense *COOLAIR* transcription facilitates non-B-DNA secondary structure formation on the template strand. In wild type *Arabidopsis*, FCA and FY-mediated R-loop resolution destabilizes the non-B-DNA structure, but the ability of the structure to transiently slow the replisome allows recruitment of SDG26 and FLD leading to propagation of silenced chromatin at the *FLC* locus. In FCA- or FY-deficient *Arabidopsis*, the R-loop is not resolved, the DNA structure persists, fork progression is stalled and epigenetic information is lost as the FLD-mediated chromatin silencing is not propagated, leading to a late flowering phenotype (Baxter et al. 2021).

### Direct detection of fork stalling at non-B DNA structures in cells

The traditional approach for directly monitoring fork stalling in cells is to use 2-dimensional electrophoresis (Brewer and Fangman 1987). This works well in budding yeast for detecting stalling at structured DNA located on a replicating plasmid (Krasilnikova and Mirkin 2004) or in the genome (Kim et al. 2008; Viterbo et al. 2016). The analysis is facilitated by the small size of the genome, well-defined replication origins and the tightly synchronous entry into S phase that can be achieved with release from alpha-factor mediated G1 arrest. In mammalian cells the technique is more challenging as capturing sufficient forks traversing a given genomic fragment to allow visualization is hampered by lack of all the features that make the method attractive in yeast. However, the approach has been successfully employed either by concentrating replication intermediates using BND-cellulose (Benzoylated Naphthoylated Diethylaminoethyl cellulose) (Mesner et al. 2006) or by using plasmids (Follonier et al. 2013). Visualization of replication tracts in combed DNA fibers isolated from cells after pulsing with halogenated nucleoside can be combined with fluorescence *in situ* hybridization (FISH) to monitor site specific fork stalling in a locus containing a repeat has been successfully employed at telomeres (Sfeir et al. 2009) and in the human *FXN* locus (Gerhardt et al. 2016). It has also recently been combined with antibody-mediated G4 detection (Stroik et al. 2020). However, this approach is somewhat akin to finding a needle-in-a-haystack and is thus very time consuming. It also offers rather limited resolution.

An interesting method for monitoring replication delays *in vivo* in real time developed by the Aharoni group uses *lacO* and *tetO* arrays knocked into a defined genomic location near a budding yeast origin of replication. The arrays are bound distinctively by fluorescent LacI and TetR molecules and, when the arrays are replicated, the fluorescence intensity doubles (Dovrat et al. 2018). The delay between signal increase of the two arrays indicates the time taken for the replisome to pass between them and thus provides an approach to monitor, in a living cell, any delay caused by a structure-forming sequence inserted between the two arrays (Dahan et al. 2018).

More recently, super-resolution optical microscopy has begun to allow visualization of the composition of replisomes in cells and to observe the spatial relationship of individual components (Yin and Rothenberg 2016). This approach has allowed the direct confirmation that G4s can indeed fold between the CMG helicase and DNA polymerases and that this is more frequent in cells lacking the FANCJ helicase (Lee et al. 2021).

## The initial encounter of the replisome with DNA secondary structure

To understand how the replisome responds to DNA secondary structures, it is helpful to first consider how the replication machinery encounters them, summarized in Figure 1.

### Encounters between the replisome and preexisting leading strand structures

Pre-formed non-B structures will be encountered by the replisome, particularly in transcriptionally active regions of the genome. Although we have considered this question in a recent review (Lerner and Sale 2019), new data on the structure and behavior of the eukaryotic replisome is emerging rapidly.

During processive replication, the eukaryotic replicative CMG helicase tracks on the leading strand. The lagging strand is displaced from the central pore and takes a path round the body of the helicase. While leading strand structured DNA could potentially be disrupted by the CMG helicase itself, there is currently limited evidence that this happens. Indeed, there is interesting genetic evidence that, in *C. elegans* lacking the major G4 helicase DOG-1 (FANCJ), a folded G4 can persist through multiple cell cycles giving rise to a lineage of animals with recurrent deletions at the site of the structure, suggesting that CMG might be able to pass the structure without its resolution (Lemmens et al. 2015). However, the diameter of many G4s at 2.4 – 2.8 nm or more (Do et al. 2011; Heddi and Phan 2011; Amrane et al. 2012; Meier et al. 2018) is larger than the narrowest point of the CMG helicase channel at ~ 1 nm. G4s should thus stall the helicase and recent experiments in *Xenopus* egg extracts suggests that this is indeed the case (Sato et al. 2021). The mode of CMG stalling in these experiments is informative and consistent with initial arrest of unwinding at the border of the structure and then with the structure being somehow accommodated within the 5′ tier of CMG. Whether this involves a structural rearrangement of CMG and/or of the structure remains to be determined, but the G4 ligand PhenDC3 prevents this from happening and leaves CMG stalled at the border of the structure (Sato et al. 2021). Continued unwinding requires one of three possibilities: the CMG helicase is removed from the DNA and reinstalled downstream of the structure; the CMG helicase remodels and traverses the structure; or the structure is unwound relieving the impediment to the helicase. Removal of CMG at DNA protein crosslinks now seems not to occur (Sparks et al. 2019a). As discussed above, direct threading of many DNA structures through the helicase seems unlikely without some structural reorganization of the complex. It is possible that such remodeling would resemble that which allows CMG to slide past DNA-protein crosslinks (Sparks et al. 2019a), a similarly bulky obstacle. This remodeling likely involves ring opening similar to that proposed during helicase activation, with opening of the interface between MCM2 and MCM5 to allow extrusion of the lagging strand (Bochman and Schwacha 2008; Fu et al. 2011; Samel et al. 2014; Noguchi et al. 2017). However, in the fully assembled CMG, the MCM2/5 gate is occluded by the GINS complex (Georgescu et al. 2017) making it less likely to be the interface that opens in the context of a traverse mechanism. A distinct mode of helicase ring opening, which depends on MCM10, has been proposed to explain the ability of the helicase to run on from single onto duplex DNA (Langston et al. 2017; Wasserman et al. 2019), and this may also be used for traverse of structures on the leading strand. Traverse of a DNA-protein crosslink is facilitated by unwinding of DNA downstream of the lesion mediated by the DNA helicase RTEL1 (Sparks et al. 2019a). An analogous role for DHX36 has recently been proposed for CMG bypass of a G4 (Sato et al. 2021), highlighting an additional role for this helicase in replicating G4s independent of its ability to unwind them.

In addition to the potential traverse mechanism, CMG helicase progression at secondary structures is likely to be facilitated by unwinding or melting of structures ahead of the helicase. While this could be achieved simply through the ability of specialized helicases to recognize structures directly, exemplified by DHX36 binding to G4s (Giri et al. 2011), recent evidence suggests that the core replisome itself may also be able to recognize DNA secondary structures. The core repli-some component Timeless contains a C-terminal myb-like zinc finger DNA binding domain (DBD), very similar to that found in the telomeric G4 binding protein Rap1 (Traczyk et al. 2020), which preferentially binds G4 structures over duplex or ssDNA (Lerner et al. 2020). The Timeless DBD may form a module with an adjacent PARP1-interacting domain (PBD) (Xie et al. 2015) to recognize secondary structures directly or through an intermediate interaction with PARP1, which can also bind G4s (Soldatenkov et al. 2008; Edwards et al. 2020). Timeless also interacts with DDX11 (Leman et al. 2010; Calì et al. 2016; Cortone et al. 2018; Lerner et al. 2020), a Fe-S 5′-3′ helicase capable of unwinding a number of DNA structures including G4s (Wu et al. 2012; Bharti et al. 2013) and triplexes (Guo et al. 2015). This suggests a mechanism to couple structure recognition by the replisome with their resolution. Recent data on the position of both the yeast Timeless homologue, Tof1, and human Timeless within the reconstituted core replisome clearly place it ahead of the MCM helicase (Baretić et al. 2020; Jones et al. 2021). Although, the C-terminus of Timeless is not mapped in either structure, the linker joining the visualized N-terminus with the C-terminal DBD and PBD domains is not long enough to allow an interaction with the unwound ssDNA behind the fork. Rather, any interaction is more likely to take place with preformed DNA structures ahead of the fork.

### Lagging strand structures

Intrastrand DNA structures formed on the lagging strand should pass around the CMG helicase, and this is observed in *Xenopus* egg extracts (Sato et al. 2021). Since the double helix would be already melted at the structure, as the helicase approaches, the point of strand separation might be expected to move from the strand separation pin, created by F285 of MCM7 in the N-terminal entry channel of CMG (Jones et al. 2021), to some distance beyond the entry channel of the helicase. Speculatively, this may alter the path of the lagging strand and thus change the interaction of the incoming duplex DNA with Timeless and Tipin, potentially facilitating the detection and resolution of associated secondary structure by proteins at the front of the CMG helicase, including by Timeless itself (Lerner et al. 2020). It would also potentially create a ssDNA ‘landing pad’for 3′-5′ G4 helicases such as DHX36 or BLM. Importantly, RPA is constitutively loaded onto the lagging strand and, as discussed below, this will play a significant role in helping destabilize secondary structure.

### Generation of structured DNA by the replisome

The CMG helicase, like other molecular motors that split the DNA strands, will generate topologically stressed ssDNA in its wake (Keszthelyi et al. 2016) that will favor non-B-DNA structure formation. Indeed, the existence of structures between the helicase and polymerase is now supported both by genetic experiments (reviewed in Šviković and Sale 2017) and direct observation (Lee et al. 2021). Since the lagging strand will be rapidly coated with RPA, it might be expected that *de novo* structure formation following CMG unwinding would be limited. However, there is no evidence the exposed leading strand is similarly bound by RPA before it enters the catalytic site of the polymerase, unless there is significant uncoupling between the two (Zou and Elledge 2003; Byun et al. 2005). Key to understanding the opportunities for structure formation behind the CMG helicase is the path that the DNA takes between the exit channel of the CMG to the catalytic center of the replicative polymerase, usually Pol å. Although current structures of the yeast and human replisome have revealed much about the detailed organization of the constituent proteins, the path of the leading strand is not yet clear. Further, the catalytic core in the C-ter-minus of the Pol2 subunit of Pol å is not yet resolved in the context of the intact replisome (Jones et al. 2021). Nonetheless, a recent structure of the isolated yeast Pol å holoenzyme has shown the non-catalytic and catalytic modules of the Pol2 subunit adopt a rigid linear conformation (Yuan et al. 2020), which can be modeled into cryo-EM structures of the yeast and human repli-somes (Yuan et al. 2020; Jones et al. 2021). If it is assumed that this conformation of Pol å represents the active form of the enzyme, then the distance between the exit pore of the CMG helicase and the catalytic center of Pol å would be in the order of 100 – 140 Å. The equates to approximately 33 – 47 bases of ssDNA DNA if completely denatured (Glass and Wertz 1980), which would allow for formation of many types of structure, for instance G4s, in the wake of the CMG even in the absence of pathological uncoupling of the helicase and polymerase induced by polymerase stalling (Figure 3).

However, the degree of fork uncoupling caused by differences in the rate of unwinding and DNA synthesis likely mean that the amount of ssDNA exposed also varies continuously, in turn modulating the opportunity for secondary structure formation. Consistent with this idea, as noted above, increasing uncoupling with DNA synthesis inhibitors increases the likelihood of G4 formation (Papadopoulou et al. 2015; Lee et al. 2021). Under these circumstances, sufficient ssDNA may be exposed to allow RPA deposition along with subsequent recruitment of factors including ATRIP, RAD18 and PrimPol (Zou and Elledge 2003; Davies et al. 2008; Guilliam et al. 2017).

It is also the case that the composition of the replisome is not fixed. The nascent replisome employs Pol δ on both leading and lagging strand immediately after origin firing before switching to use Pol ε during processive replication (Aria and Yeeles 2018; Garbacz et al. 2018). Pol δ is also deployed on the leading strand during restart of a replisome stalled at DNA damage (Guilliam and Yeeles 2020). The extent to which these different replisome compositions affect the potential for secondary structure formation remains to be explored but may predict a different burden of and response to secondary structure forming sequences in, for example, the immediate vicinity of a replication origin.

## Replicative tolerance of non-B-DNA structures

Irrespective of whether a pre-formed structure on the leading strand is bypassed by the CMG helicase or whether it forms *de novo* in the newly unwound DNA behind the CMG helicase, it will then meet the DNA polymerase and potentially stall it. This can lead to the generation of further ssDNA DNA due to the continued unwinding by the CMG helicase (Walter and Newport 2000; Pacek and Walter 2004; Byun et al. 2005). Unchecked, this could promote further secondary structure formation, potentially exacerbating the block to DNA synthesis. However, while arrest of DNA synthesis by DNA damage does not lead to immediate cessation of DNA unwinding by the CMG helicase it does appear to slow it, at least in the *in vitro* reconstituted yeast replisome (Taylor and Yeeles 2018; 2019). Thus, the helicase appears to be able to ‘sense’ that the polymerase is obstructed and to modulate its unwinding rate in response. The exact molecular mechanism of this remains unclear, but likely involves the fork protection complex (Timeless/Tipin/Claspin), which plays a key role in controlling replisome progression in response to replication stress (Katou et al. 2003; Unsal-Kaçmaz et al. 2007).

The mechanisms that allow replication forks to circumvent DNA damage that blocks DNA synthesis are collectively known as DNA damage tolerance. These mechanisms have been discussed extensively elsewhere (Ashour and Mosammaparast 2021). However, the extent to which they also operate at DNA structural impediments remains relatively poorly explored. We will next look at the main fork responses to DNA damage, direct bypass, repriming and fork reversal (Figure 3), and consider the contribution that these processes make to the replication of structural impediments.

### Direct replication: the role of the replicative polymerases

DNA secondary structures, including A-rich bent DNA, hairpins and G4s, can stall or pause replicative DNA polymerases at the boundary of the structure (Woodford et al. 1997; Kamath-Loeb et al. 2001; Shah et al. 2010). However, it is becoming evident that polymerases can also help to remodel non-B-DNA structures, potentially destabilizing them and facilitating their replication (Shah et al. 2010; Murat et al. 2020). The mechanism by which a polymerase can destabilize a structure depends on the latter’s topology and thermal stability (Takahashi et al. 2017) and this is reflected in the transience or permanence of the barrier the structure presents to DNA synthesis (Murat et al. 2020). Thus, polymerization through the simplest structure, a hairpin, only requires melting of the base pairs at the base of the structure for the polymerase to be able to make progress in a manner that is not dependent on the overall thermal stability of the structure. Of course, overall thermal stability will also depend on hairpin length, which in turn influences the overall kinetics of synthesis through such sequences (Murat et al. 2020). In the case of more complex structures such as i-motifs, polymerase access is more comprehensively inhibited through reduced breathing of the terminal base pair and steric hindrance of the polymerase by the structure itself (Takahashi et al. 2017).

An underexplored phenomenon is how polymerases become trapped within the body of a structure-forming repeat. Our recent high-throughput study of polymerase behavior in short tandem repeats (Murat et al. 2020) reveals that polymerase stalling does not occur at the boundary of a structure-forming repeat, as may be expected intuitively, but some way into the sequence. This suggests that the act of DNA synthesis through the structure is remodeling it and leading to trapping of the polymerase. Further, kinetic analysis of polymerase progress suggests that, in many cases, the stall is not static and the polymerase is gradually able to edge its way forward. This behavior is associated with a significantly greater risk of mutagenesis by the polymerase (Murat et al. 2020). The extent to which this polymerase-determined error-prone replication contributes to the increased frequency of base substitutions seen in non-B-DNA sequences *in vivo* (Du et al. 2014; Georgakopoulos-Soares et al. 2018; Guiblet et al. 2021) remains to be explored.

### Direct replication: the role of specialized polymerases

When replicative DNA polymerases stall at DNA lesions, a prominent mechanism of DNA damage tolerance is translesion synthesis (TLS), in which the stalled replicative polymerases are replaced with one or more specialized polymerases that can directly replicate lesions, albeit with a mutagenic penalty (Sale 2013). Eukaryotic TLS is carried out by the Y-family polymerases Polη, Poli, Polκ and REV1 (Sale et al. 2012) and the B-family polymerase Polζ (Martin and Wood 2019), although not all these enzymes are present in all organisms. Somewhat surprisingly, several lines of evidence point clearly to the TLS polymerases playing a role in the replication of a range of non-B structure-forming DNA sequences, even though there is no formal DNA ‘lesion’ (Hile and Eckert 2008; Bétous et al. 2009; Sarkies et al. 2010; Hile et al. 2012), suggesting that specific biochemical properties of these enzymes may be favored at repetitive or non-B DNA templates, and indeed promote their accurate replication exemplified by the behavior of Polj at short dinucleotide microsatellites (Hile et al. 2012).

Polymerase activity can also be modulated by interaction with G4 structures. Unlike the catalytic core of the replicative Polε, both Polη and Polκ exhibit a stronger preference for binding G4 compared to non-G4 substrates (Eddy et al. 2015; 2016). Further, Polη exhibits more accurate and efficient replication proximal to G4 structures as assessed by single-nucleotide insertion assays, and retained significant activity, even when the primer was positioned directly adjacent to the first tetrad-associated guanine (Eddy et al. 2015). These observations led to a kinetic switch model, whereby the kinetic differences between the activity of Polε and Polη would lead to a switch in the polymerase deployed 2-3 nucleotides prior to a G4 structure (Eddy et al. 2015). Another recent study by the Larsen laboratory indicated that although G4s largely stalled Polη-mediated synthesis, it was possible to observe partial synthesis through the first couple of tetrad-associated guanines (Berroyer et al. 2019). Interestingly, unlike Polη, Polκ inefficiently replicates tetrad-associated guanines, although its activity when the enzyme was 2-3 nucleotides from the G4 was highest of all Y-family polymerases, indicating its primary role in replicating G4s is not through direct synthesis through the motif (Eddy et al. 2016). However, when replication of a single G4 is assessed using the *BU-1* locus assay in DT40, Polη shows no significant instability of expression and Polη shows only very minor instability (Wickramasinghe et al. 2015).

Compared with the other Y-family polymerases, REV1 is an unusual enzyme. Its catalytic activity is restricted to deoxycytidyl transfer (Masuda et al. 2001). Indeed, REV1 does not read the templating DNA base but rather incorporates C opposite an arginine residue in its own active site (Nair et al. 2005). Both *in vitro* and *in vivo* this catalytic activity is deployed against only a small subset of DNA lesions such as abasic sites and minor groove adducts (Zhang et al. 2002; Washington et al. 2004; Ross and Sale 2006; Nair et al. 2008; Wiltrout and Walker 2011). Nonetheless, REV1 plays a central role in DNA damage tolerance and this is dependent on interactions with PCNA, the other Y-family TLS polymerases and with Pol ζ, mediated by its C terminus (Guo et al. 2003; Ross et al. 2005).

**Figure 4:**
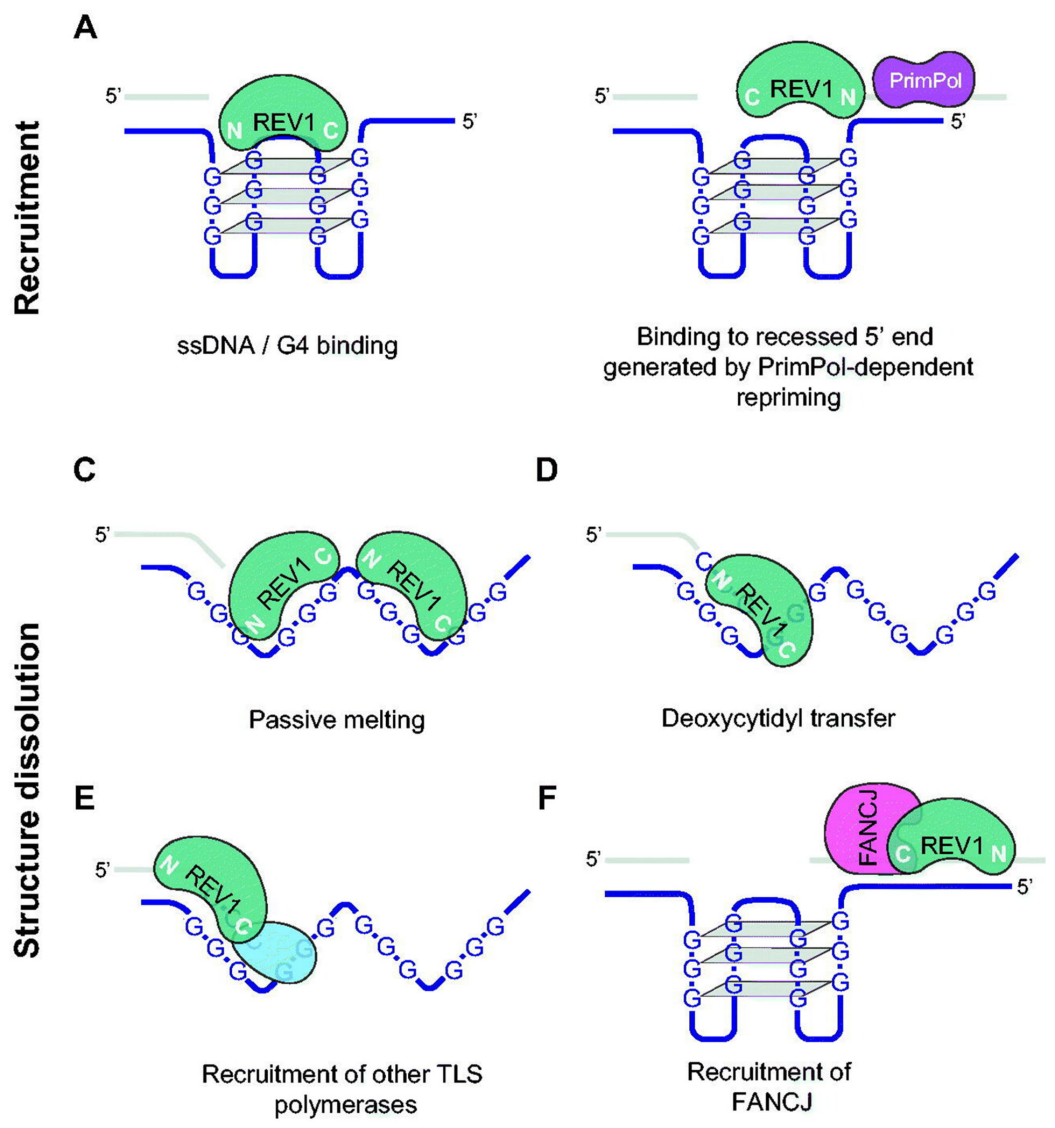
The recruitment to and resolution of G4s by REV1. (A) REV1 is a potent ssDNA binding protein and can bind G4s, possibly via the ssDNA loops between the dGs participating in the G quartets. (B) REV1 has also been shown to recognize recessed 5’ phosphate termini via a domain close to its N-terminal BRCT domain. Such a recessed end would be generated by PrimPol in a repriming event downstream of the structure. This could help recruit REV1 to the structure for downstream resolution. (C) REV1 can passively melt G4s and may also deploy its catalytic activity (D) to trap the dGs participating in the G4 quartets back into duplex DNA. (E) This may also allow recruitment of other TLS polymerases, which has also been implicated in structure resolution. (F) It may also promote the recruitment of the FANCJ to drive G4 unwinding. See text for references. A color version of this figure is available online.

Surprisingly, REV1 also plays a role in replication of G4s (Figure 4). REV1 is required to maintain epigenetic stability of G4-containing genes in DT40 cells and for efficient replication of episomal plasmids containing a leading strand G4 (Sarkies et al. 2010; Sarkies et al. 2012; Schiavone et al. 2014 and section above ‘Epigenetic instability arising from non-B DNA structuredependent fork stalling’). This requires the C terminus but there is also a clear contribution by the catalytic activity of the protein, leading us to suggest a model in which dC transfer may help trap partially unfolded dG runs from a G4 helping it back into duplex DNA (Sarkies et al. 2010). However, this not the full story of the role of the polymerase domain of REV1. *In vitro* REV1 exhibits only low efficiency nucleotide transfer opposite tetrad guanines of G4s (Eddy et al. 2014). Nonetheless, REV1 is also a potent ssDNA DNA binding protein (Masuda and Kamiya 2006) and it exhibits a remarkable ability to passively melt G4s, in a similar manner to RPA (Eddy et al. 2014). G4 binding and melting by REV1 depends on the vertebrate-specific insert-2 region of the transferase domain (Ketkar et al. 2021), which may be why REV1 does not appear to be involved in maintaining stability of a G4 array in budding yeast (Lopes et al. 2011).

Interestingly, the role of the C terminus of REV1 in G4 replication does not appear to significantly depend on the TLS polymerases with which it interacts (Wickramasinghe et al. 2015) leaving open the question of what role the C terminus plays in this context. A clue may come from the observation that REV1 also interacts with the G4 helicase FANCJ via a PCNA-interacting (PIP) motif in the latter, which binds rather better to the REV1 C-terminus than it does to PCNA (Lowran et al. 2019). That REV1 and FANCJ work in concert is consistent with the observation of a significant overlap in changes in gene expression in *rev1* and *fancj* DT40 (Sarkies et al. 2012). The significance of the interaction remains to be directly tested genetically, but speculatively it is possible to imagine a scenario in which the ability of REV1 to bind G4s might additionally recruit the FANCJ 5′-3′ helicase to assist, particularly at G4s that cannot be melted by the action of REV1 alone (Figure 4).

REV1 is also required to maintain the genetic stability of other structure-forming repeats, including hairpin-forming (CNG)n repeats (Collins et al. 2007) and triplex-forming (GAA)n repeats (Khristich et al. 2020). Mechanistically, how REV1 functions in maintaining these sequences is not clearly resolved, which may reflect the protein playing multiple roles as ssDNA-binding protein, dC transferase and scaffold (Figure 4). In the case of (GAA)n repeats, the instability is orientation dependent and would be consistent with the involvement of the catalytic activity in facilitating replication through a template hairpin when the replicative polymerases stalls at a template G within the structure. In the case of (CNG)n repeats, the *rev1-1* mutant (Larimer et al. 1989) provides evidence for involvement of the N-terminal of the protein. This region contains a BRCT domain and residues responsible for binding to 5′ and 3′ primer/template junctions, suggesting another potential mechanism for recruitment of the protein to sites of stalled DNA synthesis (de Groote et al. 2011). In both cases the instability of the repeat was not observed in cells lacking Pol ζ or Pol η. Conversely, Pol ζ, together with REV1, has been shown to be responsible for a significant proportion of the mutagenesis associated with short repeat sequences with structure-forming potential suggesting that not only is REV1 recruited to sites of transient replication arrest at secondary structures, but that can indeed also hand off to Pol ζ (Northam et al. 2014).

In the context of translesion synthesis, the ubiquiti-nation of PCNA at K164 plays a prominent role in recruitment and coordination of the specialized polymerases discussed above (reviewed in Sale 2012). PCNA ubiquitination also promotes recombinational error-free bypass (or template switching, discussed below) and this is mediated by extension of the initial monoubiquitin with a K63-linked polyubiquitin chain catalyzed in yeast by Rad5. However, evidence for PCNA ubiquitination playing an important role in driving recruitment of TLS polymerases to secondary structures is currently relatively thin. Budding yeast lacking the main E3 ubiquitin ligase for PCNA, Rad18, and in cells carrying a non-ubiquitinatable *pcna*K164R mutation, CAG.CTG repeats are more prone to expansion. However, this is likely a function of the error-free branch of the pathway, as it is also observed in cells lacking Rad5 (Daee et al. 2007). In contrast, contraction rates of triplex-forming GAA repeats in budding yeast are not affected by deletion of Rad18, despite requiring the catalytic activity of the TLS polymerase Rev1 (Khristich et al. 2020). Likewise, in the DT40 *BU-1* system, G4-dependent epigenetic instability is not observed in either a *pcnaK164R* mutant, in which PCNA cannot be ubiquitinated, or in *rad18*, (Sarkies et al. 2012), despite the prominent effect of disrupting REV1 (Sarkies et al. 2012; Schiavone et al. 2014). Thus, the evidence gathered so far does not point to PCNAUb-control of TLS polymerases as playing a central role in promoting processive synthesis through and genome stability at DNA secondary structures.

### Replication fork reversal and template switching

As noted above, PCNA polyubiquitination is linked to an error free mode of damage tolerance. Many years ago, a proposed mechanism for this form of damage bypass invoked fork reversal (Higgins et al. 1976), during which the stalled replication fork backs up allowing the nascent daughter strands anneal and form a fourway Holliday junction, returning the DNA lesion to double stranded DNA to allow safe repair. The mechanism has been observed in bacteria, where it is catalyzed by RecG (Whitby et al. 1993) and in yeast lacking the checkpoint kinase Rad53 (Sogo et al. 2002). However, it has only received significant attention as a mechanism in vertebrate cells in the past decade or so (Ray Chaudhuri et al. 2012; Neelsen and Lopes 2015). It is now clear that the complex rearrangements of fork architecture required for fork reversal in vertebrate cells is facilitated by an array of specialized factors. These include the translocases HLTF, SHPRH, the putative homologues of budding yeast Rad5, SMARCAL1, and ZRANB3 which reverse the fork, fork protection factors such as RAD51, BRCA1 and BRCA2 and finally helicases which allow resolution of the reversed fork and restart of replication, such as DNA2, WRN and RECQ1 (Quinet et al. 2017). Fork reversal has been observed by electron microscopy during replication of (GAA)n repeats in an SV40-based plasmid (Follonier et al. 2013). However, its importance in responding to and removing DNA secondary structure remains somewhat unclear and may vary depending on the nature of the sequence and the sequence’s propensity to form a structure. Thus, budding yeast cells lacking the Rad5-dependent pathway of DNA damage tolerance have been reported to exhibit a greater rate of expansions at (CAG)n repeats (Daee et al. 2007) but at (GAA)n repeats expansions are reduced (Shishkin et al. 2009). Likewise, expansions are reduced at a (ATTCT)n repeat, which is not capable of forming secondary structure in Rad5D cells (Cherng et al. 2011).

Nonetheless, it remains unclear what advantage fork reversal provides in a context where there is no DNA damage. It is possible that the forces required to remodel the fork during the reversal reaction would also destabilize many non-B DNA structures effectively allowing replication to resume.

### Repriming

Repriming beyond a replication impediment is a conceptually straightforward way to alleviate stalled DNA synthesis that confines the impediment to a short gap, which can be filled post-replicatively. Repriming by the core replisome primase (PRIM1) could in theory bypass replication impediments caused by structured DNA and would be a natural response on the lagging strand, where priming is in any event frequent due to Okazaki fragment synthesis. It has however been observed that Pif1 deletion leads to retarded replication of a G4-containing lagging strand in yeast, so replisome repriming activity alone is not sufficient to ensure efficient replication of lagging strand structured DNA (Dahan et al. 2018). In contrast, the contribution of core replisome repriming on the leading strand to replicative tolerance of structured DNA is likely small if parallels can be drawn with the behavior of the reconstituted yeast replisome, which exhibits very inefficient leading strand repriming beyond replication impediments caused by a range of damaged bases (Taylor and Yeeles 2018; 2019).

Many eukaryotes possess a second DNA primase, PrimPol (Bianchi et al. 2013; García-Gómez et al. 2013; Wan et al. 2013), which belongs to the archaeo-eukaryotic primase (AEP) superfamily (Iyer et al. 2005). PrimPol is found in a range of single-celled and multicellular eukaryotes (plants, animals and protists included), although there are some prominent exceptions including the fruitfly *Drosophila melanogaster* and nematode worm *Caenorhabditis elegans* (Bainbridge et al. 2021). PrimPol reprimes after a variety of DNA damage lesions, allowing continued DNA replication in the presence of DNA damage (Bianchi et al. 2013; Mourón et al. 2013; Keen et al. 2014). Analogous to its role in DNA damage tolerance, PrimPol also reprimes beyond non-B DNA structures. It can reprime past G4s *in vitro*, facilitated by its ability to bind the structure (Schiavone et al. 2016). In DT40 cells, PrimPol plays a very prominent role in repriming at G4s and triplex-forming short (GAA)n repeats (Schiavone et al. 2016; Šviković et al. 2019). This requires not only the catalytic activity and zinc finger domain, but also the first of two RPA-binding domains, consistent with PrimPol recruitment to RPA-coated ssDNA formed around the structures (Guilliam et al. 2017; Šviković et al. 2019). How is the remaining structure-containing gap resolved? The most straightforward mechanism would be structure resolution by specialized helicases (Table 1) followed by gap filling. Recent observations on PrimPol-mediated repriming following bulky DNA damage adducts show that the gaps are substrates for resection, RAD51 loading and homologous recombination (Piberger et al. 2020) and for REV1/ Polf-dependent TLS, especially when recombination is inactivated, for instance by inactivation of BRCA2 (Taglialatela et al. 2021; Tirman et al. 2021). However, the extent to which such post-replicative events are induced by DNA structures remains unclear.

## Mechanisms that help the replisome through non-B DNA secondary structures

While some non-B-DNA structures can be overcome by the replisome directly, there remain circumstances in which it requires assistance through suppression or removal of structures to prevent them being an obstacle to ongoing fork progression. The processes that provide this assistance can broadly be considered as passive or active. Passive mechanisms rely on the fact that formation of structure requires free ssDNA and proteins which occupy DNA can suppress or reverse structure formation. Active mechanisms, on the other hand, use ATP-driven enzymatic processes to remove structured DNA. However, as will become apparent, the line between passive and active mechanisms can be blurred with many ‘active’ enzymes also playing a ‘passive’ role in controlling structure formation.

### Chromatin

Packaging DNA within chromatin can restrict the formation of some DNA secondary structures and may therefore be considered as a potential mechanism to limit the number of pre-formed structures encountered by the replisome. There is, however, a complex relationship between DNA packaging within chromatin and secondary structures. The sequence requirements for secondary structure formation generally do not overlap with those that favor nucleosome formation (Struhl and Segal 2013). Several potentially structure-forming repeat sequences, e.g. [CTG]_n_ (Wang et al. 1994; Wang and Griffith 1995) have strong positive effects on nucleosome positioning. Others e.g. [CCG]n (Wang et al. 1996), [(G/C)3NN] (Wang and Griffith 1996) and poly(dA:dT) or poly(dG.dC) (reviewed in Struhl and Segal 2013) proficiently exclude nucleosomes. These effects are driven by the energetics of DNA bending around the nucleosome. Structure formation, including Z-DNA and cruciforms exclude nucleosomes (Nickol et al. 1982; Nickol and Martin 1983; van Holde and Zlatanova 1994; Wong et al. 2007), which interact preferentially with B-form DNA. Early genome wide analyses identified that predicted G4 structures were enriched outside regions of high nucleosome occupancy in *S. cerevisiae*, *C. elegans* and in humans (Hershman et al. 2008; Halder et al. 2009; Wong and Huppert 2009). More recent techniques, including G4 ChIP-seq (Hänsel-Hertsch et al. 2016), permanganate/S1 nuclease footprinting (Kouzine et al. 2017) and nascent strand sequencing (NS-seq) (Foulk et al. 2015) have confirmed an inverse relationship between DNA structure formation and nucleosome occupancy. Many of these sites of structure formation are associated with precise positioning of flanking nucleosomes. These sites often appear to have potential for gene regulation (Hänsel-Hertsch et al. 2016; Kouzine et al. 2017) or replication origin specification (Foulk et al. 2015), although the relationship between structure-forming capacity and replication origin efficiency is not that strong (Guilbaud et al. 2022). Thus, is possible that structure forming sequences may, in some cases, be selected for their ability to promote nucleosome positioning or repositioning with the corollary that such sites will also be at more significant risk of fork stalling.

If structure formation inhibits nucleosome placement, mechanisms to ensure full nucleosome occupancy in ‘at risk’ regions of the genome could prevent DNA structure formation. An example of this principle may be seen in the work of the chromatin remodelers ATRX and DAXX at telomeres. ATRX binds to G4s (Law et al. 2010) and through cooperation with its interaction partner, the histone chaperone DAXX, can load the histone variant H3.3 onto telomeric DNA (Lewis et al. 2010). Increased telomeric recombination is a feature of tumors which rely on the alternative lengthening of telomere (ALT) pathway to maintain telomere length independent of increased telomerase expression. Mutations in the ATRX/DAXX/H3.3 system are a common feature of such tumors (Heaphy et al. 2011). Restoration of ATRX activity in ATRX-deficient ALT cell lines leads to suppression of the ALT phenotype, in a process that is dependent on the activity of DAXX and increased levels of telomeric histone H3.3 and that is suppressed by G4 stabilization (Clynes et al. 2015).

Thus, the increased telomeric recombination which underlies the ALT phenotype could be driven by the persistence of G4 secondary structures in the telomeres that result from altered chromatinisation in the absence of ATRX activity. Supporting this, and a more general genomic role for ATRX, recent studies have demonstrated that ATRX deficient cell lines exhibited increased G4 formation in newly synthesized DNA, replication stress and DNA damage and that they accumulate copy number alterations at a greater rate than WT cells (Wang et al. 2019; Teng et al. 2021). The protective effect of ATRX is lost in mutants that do not support its role in H3.3 incorporation into chromatin or by mutations within the predicted helicase domain. However, to our knowledge, ATRX has not yet been shown biochemically to exhibit helicase activity.

### Single strand DNA binding proteins

In double-stranded DNA, folding of structured DNA is in competition with formation of a B-DNA form duplex, lessening the likelihood of its formation. We discussed above processes that can drive ssDNA formation and that could therefore increase the chance of DNA secondary structure folding. In terms of replication, the discontinuous replication of lagging strand DNA intrinsically increases exposure of ssDNA compared to the leading strand, so it might be expected that the lagging strand template would be more prone to replication-associated structure formation than the leading strand. In reality however, the exposed lagging strand ssDNA is rapidly coated by the heterotrimeric protein complex RPA, sequestering ssDNA and restricting its availability to form DNA secondary structures. RPA is the major eukaryotic single stranded binding protein with key roles in ensuring correct processing of ssDNA during DNA replication, DNA repair and DNA recombination (Caldwell and Spies 2020). RPA has been shown to restrict formation of multiple types of non-B DNA structures when binding to ssDNA templates, including hairpins and G4s (Chen et al. 2013; Audry et al. 2015). It is now clear that RPA binding to ssDNA is not only able to prevent structure formation, but is able to promote melting of folded structures, including a range of single G4s and also contiguous G4s as found in telomeric DNA (Salas et al. 2006; Fan et al. 2009; Ray et al. 2013; Lancrey et al. 2018). The mechanism by which RPA binds and unfolds G4s is becoming increasingly well understood, with the current model suggesting RPA unfolds G4s through a sequential binding mechanism with 5′ to 3′ directionality (Safa et al. 2014; 2016), analogous to its binding to unstructured ssDNA (Kolpashchikov et al. 2001). Through this ability to unfold structured DNA, RPA binding likely suppresses formation of many DNA secondary structures on the lagging strand. However, this may not extend to more stable G4s with deeper quartet stacks and short loops (Ray et al. 2013), which may reflect how RPA initially binds G4s either directly on the ssDNA ‘loops’ of the structure or indirectly on adjacent ssDNA. Mechanistically, the initial binding of RPA to ssDNA is thought to be mediated by DNA binding domain A and B in RPA70. Each DBD has a footprint of 3 nt and their contacts are separated by 2 nucleotides. Thus, Ray et al. proposed that initial binding via DBA-A may be sufficient to initiate the process of denaturation of the G4 to allow sequential binding of DNA-B and then DBD-C and D as the structure denatures (Ray et al. 2013). Such a model would explain why RPA preferentially melts G4s with longer loops and fewer stacks. This idea is supported by a recent biochemical study that examined the ability of RPA to alleviate stalling of DNA polymerase δ at a range of structured sequences in during a primer extension assay (Sparks et al. 2019b). RPA was able to promote replication through hairpins and weak G4s but not through more thermodynamically stable structures for which the assistance of the Pif1 helicase was required to allow DNA synthesis to proceed. The importance of RPA in preventing genetic instability during replication of structure-forming sequences is underscored by the effect of mutations in the OB fold of DBD-A. The *rpa1-D223Y* mutant in S. pombe exhibits impaired telomere replication and accumulates telomere defects (Audry et al. 2015). Likewise, both S. pombe rpa1-D223Y and S. cerevisiae rpa1-D228Y (the equivalent mutation in budding yeast) exhibit genetic instability associated with replication of the G4-containing human microsatellite sequences (Maestroni et al. 2020).

In the context of telomeres, Pot1 is a related OB-fold containing protein that, with its interaction partner TPP1, controls G4 formation within the telomeric repeats. Like RPA, Pot1 can melt telomeric G4 structures, but in contrast to RPA does so in a 3′ to 5′ direction (Hwang et al. 2012; Chatain et al. 2021). In conjunction with its binding partner TPP1, it is able to slide along telomeric repeat DNA, which may contribute to the ability of this complex to increase the processivity of telomerase. Interestingly, Pot1 is not able to completely substitute for the role of replication associated RPA in countering telomeric G4 formation as *rpa1-D223Y* cells exhibit reduced Pot1 recruitment and telomeric replication defects, which can be rescued by overexpression of the Pif1 helicase (Audry et al. 2015).

### Helicases acting at non-B DNA structures during replication: active and passive mechanisms

The activity of RPA and POT1 in countering secondary structure establishes a principle of passive structure dissolution that is emerging as a feature of many proteins that are also capable of actively contributing to structure resolution. For instance the Y-family polymerase REV1 (Eddy et al. 2014) and several of the specialized helicases, for example BLM (Budhathoki et al. 2014) and DHX36/RHAU (Yangyuoru et al. 2018; Chen et al. 2018a; 2018b), significantly depend on passive mechanisms for destabilizing secondary structures that mediate structure unwinding.

In this regard, the G4 unfolding mechanism of DHX36 is of particular interest as it illustrates a complex interaction between ATP-independent and ATP-dependent actions that culminate in the stepwise melting of parallel G4s. DHX36 binds both RNA and DNA G4s very tightly (Creacy et al. 2008; Giri et al. 2011), mediated by the cooperative binding of the DHX36-specific motif, DSM, and an OB fold domain, which recognize the planar face of the top quartet and the conformation of the backbone of one of the G-tracts respectively, with an additional contribution from a flexible loop in the RecA2 domain (Lattmann et al. 2010; Meier et al. 2013; Heddi et al. 2015; Chen et al. 2018b; Hossain et al. 2021). Single molecule and structural analysis of the *Drosophila* enzyme suggested that initial binding to the G4 leads to relatively minor but important disruption of the G quartets within the G4, with ATP hydrolysis leading to its more radical destabilization and melting due to translocation of the enzyme along the 3′ tail, in a 3′ to 5′ direction, into the structure (You et al. 2017; Chen et al. 2018b). A study of the *Bos taurus* enzyme in complex with the G4 from the *MYC* promoter advanced a slightly different model in which the enzyme is able to employ the binding energy of interaction with the G4 to exert a pulling force that is able to destabilize the structure one base at a time, with ATP hydrolysis being needed for substrate release (Chen et al. 2018a). In both models, however, important destabilization of the G4 takes place passively, but is coupled to an ATP hydrolysis step.

A significant additional body of helicases play key roles in facilitating replication of non-B DNA structures, a topic covered in detail in a number of recent reviews (Mendoza et al. 2016; Lerner and Sale 2019). Some of their key features are summarized in Table 1. We next focus on the emerging evidence for the distribution of helicase recruitment and function around the fork, and the cooperation between the helicases and with other mechanisms that prevent structured DNA from impeding replication.

## Specificity and redundancy in mechanisms for replicating non-B DNA structures

DNA structures exhibit significant conformational and thermodynamic diversity, even within a broad class, such as G4s, which we will use to illustrate the key points in this last section. Similar arguments can be applied to other structures (see Table 1). It might be expected therefore that the selection of mechanisms to disrupt structures may be influenced by specific features of those structures. For instance, the number of G tetrads, parallel vs. antiparallel topology and loop size of G4s. While enzymes such as PIF1 and BLM appear to be active to varying extents on a wide range of G4 structures (reviewed in Lerner and Sale 2019), others appear to favor specific conformations or molecular configurations. For example, DDX36/RHAU exhibits a preference for parallel G4s (Tippana et al. 2016) while DDX11 favors anti-parallel G4s (Wu et al. 2012). The FANCJ helicase is able to unwind unimolecular G4s, which defeat the closely related helicase DDX11, which in turn is able to act on intramolecular G4 structures (Bharti et al. 2013) and triplexes (Guo et al. 2015).

There is similar evidence of preferential use of ssDNA binding proteins on structures with certain features. The ability of RPA to promote G4 melting is most effective if the G4 has fewer quartet stacks and longer loops (Qureshi et al. 2012; Ray et al. 2013), which may simply reflect limits on the ability of RPA to drive more thermodynamically stable structures back to duplex. Likewise, REV1 exhibits preferential binding to parallel G4 structures (Ketkar et al. 2021) although in cells REV1 appears to play a more prominent role at thermodynamically weaker G4s with longer loops that adopt antiparallel or hybrid conformation (Schiavone et al. 2014). Conversely, repriming by PrimPol is more prominent during the replication of a highly thermodynamically stable, short-looped G4 (Schiavone et al. 2016).

The context in which a DNA structure appears will also play a key role in determining the factors which interact with it. The general requirement of an ssDNA landing pad to allow helicase loading may mean that the helicase(s) that ultimately act on a specific structure may in part be determined by the availability of ssDNA 5′ or 3′ to the structure favoring deployment of helicases of the appropriate polarity. A specific example of this is seen with the BLM helicase, which requires 3′ ssDNA to be able to unwind a G4 substrate (Sun et al. 1998).

The observation of such biases in the activity of different factors does not mean that structures are strictly processed by individual mechanisms. Indeed, there is evidence for the expected redundancy, as observed between BLM and WRN in the replication of G4s at the *BU-1* locus in DT40 (Sarkies et al. 2012). Similarly, loss of both of the G4 helicases FANCJ and DDX11 leads to greater G4-dependent expression instability of the *BU-1* locus than is seen following loss of each individually, suggesting both contribute independently to replication of this structure (Lerner et al. 2020).

Thus, the extent to which groups of structure resolution mechanisms are organized into pathways or modules, needs to be further investigated. As for damage tolerance (Sale 2012), significant redundancy in the mechanisms deployed is likely to be essential for ensuring that non-B-form structural impediments to replication are dealt with promptly and robustly. Clues are emerging that the mechanisms deployed depend not only on the form of the secondary structure, but also the context in which it is found and that these mechanisms may ultimately prove as intricate and complex as the more widely studied DNA repair pathways.

## Figures and Tables

**Table 1:** Summary of helicases shown biochemically to be involved in the resolution of non-B-form DNA.

Helicase	Polarity	Substrates	Superfamily (subfamily)	Notes
PIF1	5′-3′	Parallel and antiparallel G4s (Mendoza et al. 2015); DNA hairpins (Li et al. 2016; Sparks et al. 2019b)	SF1	Binds to 3′ ssDNA-dsDNA junctions (Zhou et al. 2014). Loss leads to genomic instability associated with leading strand G4-forming arrays in yeast (Ribeyre et al. 2009; Lopes et al. 2011) but also delayed fork progression when G4s are present on the lagging strand (Dahan et al. 2018). Present in the mitochondria, where it also contributes to G4 suppression (Lahaye et al. 1991; Cheng et al. 2007).
DNA2	5′-3′	Telomeric G4s (Masuda-Sasa et al. 2008); DNA hairpins (Lee et al. 2013)	SF1	Also possesses nuclease activity at G4s, with DNA2 deficiency leading to defective telomere replication in mouse cells (Lin et al. 2013). The relative contribution of the helicase and cleavage activities is as yet unclear. Both the endonuclease and helicase activities of DNA2 contribute to the removal of hairpin structure-forming flaps during Okazaki fragment processing (Lee et al. 2013).
FANCJ	5′-3′	Parallel and antiparallel G4s (Bharti et al. 2013), triplex DNA (Sommers et al. 2009); DNA hairpins (Bharti et al. 2018)	SF2 (Fe-S)	Requires a 5′ ssDNA ‘landing pad’ for efficient unwinding of G4s (Wu et al. 2008). Loss of FANCJ leads to genomic instability associated with G4-forming sequences in FANCJ-deficient human cells and *C. elegans* (Cheung et al. 2002; Youds et al. 2006; Kruisselbrink et al. 2008; London et al.2008; Koole et al. 2014) and epigenetic instability associated with replication of a G4-forming sequence in avian DT40 cells (Sarkies et al. 2012). Interacts with the TLS polymerase REV1 with which it collaborates to replicate G4s (Sarkies et al. 2012; Lowran et al. 2019).
DDX11	5′-3′	Intermolecular and intramolecular triplex DNA (Guo et al. 2015), G4s (Wu et al. 2012; Bharti et al. 2013)	SF2 (Fe-S)	Requires a 5′ssDNA tail for both triplex and G4 unwinding (Guo et al., 2015). Increased triplex formation and double strand breaks in DDX11-deficient cells (Guo et al., 2015). Interacts with the core replisome component Timeless to maintain replication through G4s (Lerner et al., 2020). Loss of DDX11 leads to epigenetic instability and DNA damage associated with G4s (Lerner et al., 2020).
RTEL1	5′-3′	Telomeric G4s (Vannier et al. 2013); DNA hairpins (Frizzell et al. 2014)	SF2 (Fe-S)	Loss of RTEL1 leads to telomeric instability further increased upon G4 stabilization (Vannier et al. 2012; 2013). RTEL1 acts to prevent expansion of hairpin-forming (CTG.CAG) trinucleotide repeats (Frizzell et al. 2014).
XPD	5′-3′	Parallel tetramolecular G4 (Gray et al. 2014)	SF2 (Fe-S)	XPD binding sites overlap with G4 motifs in human cells (Gray et al. 2014). Note: the SF2 helicase XPB, also part of TFIIH, binds but does not unwind G4s (Gray et al. 2014).
WRN	3′-5′	Triplex DNA (Brosh et al. 2001); G4s with particular specificity for telomeric G4s (Tippana et al. 2016); DNA cruciforms (Mohaghegh et al. 2001; Compton et al. 2008); DNA hairpins (Kamath-Loeb et al. 2001; Chan et al. 2012).	SF2 (RecQ)	Requires a 3′ ssDNA overhang to unwind G4s (Mohaghegh et al. 2001). Loss of WRN leads to defective replication of telomeres (Crabbe et al. 2004). Loss of WRN leads to dysregulated expression of some genes harboring G4s around their transcription start sites (Johnson et al. 2010; Sarkies et al. 2012).
BLM	3′-5′	Triplex DNA (Brosh et al. 2001), parallel and antiparallel G4s (Mohaghegh et al. 2001; Tippana et al. 2016); i-motifs (Gao et al. 2022); DNA hairpins (Wang et al. 2015); cruciforms (Mohaghegh et al. 2001).	SF2 (RecQ)	Requires a 3′ ssDNA overhang to unwind G4s (Sun et al. 1998). Binding to ssDNA adjacent to G4s can destabilize the structure passively (Budhathoki et al. 2014). Facilitates telomere replication (Drosopoulos et al. 2015) and acts to suppress recombination at G4 motifs (van Wietmarschen et al. 2018). In *C. elegans*, the BLM homologue HIM-6 restricts deletion of G-rich tracts in the absence of the FANCJ homologue (Youds et al. 2006). Loss of BLM leads to dysregulated expression of some genes harboring G4s within their transcription start sites (Johnson et al. 2010; Sarkies et al. 2012) and many more when combined with loss of WRN (Sarkies et al. 2012). A recent *in vitro* study has demonstrated the ability of human BLM to bind and unwind i-motif-forming sequences, in a process apparently independent of a requirement for ATP (Gao et al. 2022).
RECQ1	3′-5′	Telomeric G4s (Liu et al. 2021)	SF2 (RecQ)	Previous work showed RECQ1 was unable to unwind intermolecular G4s (Popuri et al. 2008), however a recent study has demonstrated the ability of RECQ1 to unwind intramolecular G4s (Liu et al. 2021). Loss of RECQ1 leads to telomere defects (Popuri et al. 2014).
RECQ5	3′-5′	G4s (Budhathoki et al. 2016)	SF2 (RecQ)	The observed G4 unfolding activity of RECQ5 is an order of magnitude less than that observed for BLM (Budhathoki et al. 2016)
DHX9	3′-5′	Triplex DNA (Jain et al. 2010); G4s (Chakraborty and Grosse 2011)	SF2 (DEAH)	Depletion of DHX9 in human cells leads to increased mutagenesis associated with a H-DNA-forming sequence in a *supF* reporter plasmid system (Jain et al. 2010).
RHAU/DHX36	3′-5′	Parallel G4s (Vaughn et al. 2005; Heddi et al. 2015); i-motifs (Gao et al. 2022)	SF2 (DEAH)	In addition to an ATP-dependent mechanism of G4 resolution (Vaughn et al. 2005), also possesses an ATP independent mechanism of structure dissolution (Yangyuoru et al. 2018; Chen et al. 2018a; Gao et al. 2022). *Bos taurus* DHX36 is able to bind and unwind i-motifs *in vitro*, with a contribution independent of ATP (Gao et al. 2022).
DDX5	N/A	Myc G4 (Wu et al. 2019)	SF2 (DEAD)	Does not require an ssDNA overhang and unfolding is independent of ATP hydrolysis (Wu et al. 2019). Other DEAD-box helicases (DDX1, DDX24, DDX42) have also been identified as G4 binders, raising the possibility that these unwind G4s by the same mechanism (Zyner et al. 2019; Zhang et al. 2021).
